# Editorial: Ca^2+^ signalling in plant biotic interactions

**DOI:** 10.3389/fpls.2023.1137001

**Published:** 2023-01-20

**Authors:** Thomas A. DeFalco, Wolfgang Moeder, Keiko Yoshioka

**Affiliations:** ^1^ Department of Biology, Western University, London, ON, Canada; ^2^ Department of Cell & Systems Biology, University of Toronto, Toronto, ON, Canada

**Keywords:** calcium, ion channels, biotic interaction, immunity, PTI

Calcium ions (Ca^2+^) serve as a universal second messenger across eukaryotes (Clapham, 2007). In the paradigm of Ca^2+^ signalling, stimuli trigger rapid changes in free Ca^2+^ concentration *via* the coordinated activities of Ca^2+^-permeable channels, pumps, and antiporters (‘encoding’) (McAinsh and Pittman, 2009). These signals can occur across cellular compartments (Resentini et al., 2021) where they are sensed *via* suites of Ca^2+^-binding sensor proteins (‘decoding’), which in turn regulate numerous downstream cellular processes, ultimately eliciting stimulus-appropriate physiological responses (DeFalco et al., 2010). Great progress has been made in the past decade in the development and deployment of new tools to monitor and visualize *in vivo* calcium signals (Grenzi et al., 2021), spurring a renaissance in the field of plant Ca^2+^ signalling.

In keeping with its evolutionarily-conserved and universal role, Ca^2+^ signalling is central to diverse aspects of plant development as well as responses to environmental perturbations (Kudla et al., 2018), including, notably, biotic stimuli (Tian et al., 2020; Köster et al., 2022; Xu et al., 2022). Ca^2+^ fluxes are among the earliest detectable responses to the perception of pathogens and pests (Yu et al., 2017; DeFalco and Zipfel, 2021) as well as symbiotic microbes (Tian et al., 2020), and as such have been a major area of focus in molecular plant biotic interaction research in recent years. Such work has led to key recent discoveries in the field of biotic interactions, including the identification of numerous Ca^2+^ channels playing roles in both cell surface and intracellular immunity (Bi et al., 2021; Bjornson et al., 2021; Jacob et al., 2021; Köster et al., 2022; Xu et al., 2022).

In this Research Topic issue, several important aspects of Ca^2+^ signalling in the context of plant biotic interactions have been advanced. Ca^2+^ signalling is a central component of many stress response pathways in plants, and Patra et al provide an overview of Ca^2+^ signalling networks in both abiotic and biotic stress contexts. One of the key downstream effectors of Ca^2+^ signalling in immunity is the transcriptional regulator CAMTA3/AtSR1, which is regulated by the central Ca^2+^ sensor calmodulin (CaM) and acts as an executor of the general stress response (Bjornson et al., 2021). Here, Yuan et al provide a detailed summary of the functions of this transcription factor in immunity, its regulation by CaM and phosphorylation, and its guarding by intracellular immune receptors.

While Ca^2+^ signalling is one of the early hallmarks in response to immune elicitors such as the bacterial flagellin-derived epitope flg22, whether such signals are altered in the context of immune priming was unknown. Eichstadt et al developed an approach to examine Ca^2+^ signals in both local and systemic leaves upon immune elicitation using the ratiometric fluorescent calcium indicator R-GECO1-mTurquoise, which allowed them to determine that immune priming does not alter rapid Ca^2+^ signalling dynamics in distal tissues.

Many Ca^2+^-permeable channels have been implicated in plant biotic responses, including members of the cyclic nucleotide-gated channel (CNGC) family (DeFalco et al., 2016). Sun et al report that CNGC2, a well-studied member of this family, contributes to the Ca^2+^ signal induced by extracellular ATP (eATP), which acts as an immune elicitor upon cellular damage in plants. Finally, while most signalling work has been performed using model plants such as Arabidopsis, Zhou et al have examined how Ca^2+^ signalling and transporters contribute to flagellin-induced immune responses in cotton (*Gossypium hirsutum*), identifying a Ca^2+^ efflux transporter as a potential regulator of defenses to Verticillium wilt.

The tools available to researchers that allow for monitoring of *in planta* Ca^2+^ dynamics continue to expand and improve (Waadt et al., 2021), allowing for ever-more complex and detailed study of Ca^2+^ signals, from organelles to single cells to whole plants. Recent insights have also expanded our understanding of the repertoire of Ca^2+^-permeable channels in plants and their potential functions in plant immunity (Köster et al., 2022; Xu et al., 2022). Such breakthroughs have opened key questions for the field, which is poised for fundamental discoveries in coming years.

## Author contributions

All authors listed have made a substantial, direct, and intellectual contribution to the work and approved it for publication.
